# Supplementary material for: Heparin-based hydrogel scaffolding alters the transcriptomic profile and increases the chemoresistance of MDA-MB-231 triple-negative breast cancer cells
Source: Biomater Sci. 2020 Feb 13;8(10):2786–96. doi: 10.1039/c9bm01481k (PMC7497406; doi:10.1039/c9bm01481k)
Supplement: Supplementary file 2 [file BM-008-C9BM01481K-s002.zip › Supplementary File 4/EGFvControl/Pathways/my_analysis.Gsea.1545200981068/HALLMARK_HEDGEHOG_SIGNALING.html]

Details for gene set HALLMARK\_HEDGEHOG\_SIGNALING[GSEA]

|  || Dataset | expr.class.cls#EGF\_versus\_CONTROL.class.cls#EGF\_versus\_CONTROL\_repos |
| Phenotype | class.cls#EGF\_versus\_CONTROL\_repos |
| Upregulated in class | CONTROL |
| GeneSet | HALLMARK\_HEDGEHOG\_SIGNALING |
| Enrichment Score (ES) | -0.40989098 |
| Normalized Enrichment Score (NES) | -1.3818817 |
| Nominal p-value | 0.0940367 |
| FDR q-value | 0.04434264 |
| FWER p-Value | 0.481 |
Table: GSEA Results Summary

  

Fig 1: Enrichment plot: HALLMARK\_HEDGEHOG\_SIGNALING      
 Profile of the Running ES Score & Positions of GeneSet Members on the Rank Ordered List

  

| PROBE | DESCRIPTION (from dataset) | GENE SYMBOL | GENE\_TITLE | RANK IN GENE LIST | RANK METRIC SCORE | RUNNING ES | CORE ENRICHMENT || 1 | CDK6 | na |  |  | 1642 | 1.309 | -0.0267 | No |
| 2 | ETS2 | na |  |  | 1829 | 1.261 | 0.0205 | No |
| 3 | RTN1 | na |  |  | 2606 | 1.093 | 0.0293 | No |
| 4 | CRMP1 | na |  |  | 2635 | 1.088 | 0.0768 | No |
| 5 | TLE1 | na |  |  | 4070 | 0.840 | 0.0399 | No |
| 6 | RASA1 | na |  |  | 4463 | 0.783 | 0.0548 | No |
| 7 | CDK5R1 | na |  |  | 5732 | 0.605 | 0.0159 | No |
| 8 | NF1 | na |  |  | 5879 | 0.587 | 0.0347 | No |
| 9 | NKX6-1 | na |  |  | 7200 | 0.422 | -0.0151 | No |
| 10 | UNC5C | na |  |  | 7415 | 0.395 | -0.0085 | No |
| 11 | NRCAM | na |  |  | 8742 | 0.241 | -0.0668 | No |
| 12 | OPHN1 | na |  |  | 9014 | 0.213 | -0.0713 | No |
| 13 | PTCH1 | na |  |  | 10340 | 0.063 | -0.1376 | No |
| 14 | NRP2 | na |  |  | 11171 | -0.023 | -0.1799 | No |
| 15 | PLG | na |  |  | 13548 | -0.322 | -0.2893 | No |
| 16 | DPYSL2 | na |  |  | 15862 | -0.660 | -0.3802 | Yes |
| 17 | PML | na |  |  | 15940 | -0.675 | -0.3537 | Yes |
| 18 | TLE3 | na |  |  | 15944 | -0.676 | -0.3234 | Yes |
| 19 | LDB1 | na |  |  | 15959 | -0.679 | -0.2936 | Yes |
| 20 | ACHE | na |  |  | 16288 | -0.755 | -0.2766 | Yes |
| 21 | L1CAM | na |  |  | 16651 | -0.846 | -0.2574 | Yes |
| 22 | CELSR1 | na |  |  | 17918 | -1.286 | -0.2655 | Yes |
| 23 | SCG2 | na |  |  | 18189 | -1.419 | -0.2156 | Yes |
| 24 | NRP1 | na |  |  | 18351 | -1.527 | -0.1552 | Yes |
| 25 | SHH | na |  |  | 18761 | -1.989 | -0.0869 | Yes |
| 26 | VLDLR | na |  |  | 18950 | -2.433 | 0.0129 | Yes |
Table: GSEA details [plain text format]

  

Fig 2: HALLMARK\_HEDGEHOG\_SIGNALING      
 Blue-Pink O' Gram in the Space of the Analyzed GeneSet

  

Fig 3: HALLMARK\_HEDGEHOG\_SIGNALING: Random ES distribution      
 Gene set null distribution of ES for **HALLMARK\_HEDGEHOG\_SIGNALING**
